# Blood transcriptome reveals immune and metabolic-related genes involved in growth of pasteurized colostrum-fed calves

**DOI:** 10.3389/fgene.2023.1075950

**Published:** 2023-02-06

**Authors:** Chenglong Li, Shuzhen Li, Chaoyun Yang, Yanling Ding, Yanfeng Zhang, Xiaowei Wang, Xiaonan Zhou, Zonghua Su, Wenxuan Ming, Ling Zeng, Yun Ma, Yuangang Shi, Xiaolong Kang

**Affiliations:** Key Laboratory of Ruminant Molecular and Cellular Breeding, School of Agriculture, Ningxia University, Yinchuan, China

**Keywords:** pasteurization, Holstein, growth traits, immunity, colostrum

## Abstract

The quality of colostrum is a key factor contributing to healthy calf growth, and pasteurization of colostrum can effectively reduce the counts of pathogenic microorganisms present in the colostrum. Physiological changes in calves fed with pasteurized colostrum have been well characterized, but little is known about the underlying molecular mechanisms. In this study, key genes and functional pathways through which pasteurized colostrum affects calf growth were identified through whole blood RNA sequencing. Our results showed that calves in the pasteurized group (*n* = 16) had higher body height and daily weight gain than those in the unpasteurized group (*n* = 16) in all months tested. Importantly, significant differences in body height were observed at 3 and 4 months of age (*p* < 0.05), and in daily weight gain at 2, 3, and 6 months of age (*p* < 0.05) between the two groups. Based on whole blood transcriptome data from 6-months old calves, 630 differentially expressed genes (DEGs), of which 235 were upregulated and 395 downregulated, were identified in the pasteurized compared to the unpasteurized colostrum groups. Most of the DEGs have functions in the immune response (e.g., *CCL3*, *CXCL3*, and *IL1A*) and metabolism (e.g., *PTX3* and *EXTL1*). Protein-protein interaction analyses of DEGs revealed three key subnetworks and fifteen core genes, including UBA52 and RPS28, that have roles in protein synthesis, oxidative phosphorylation, and inflammatory responses. Twelve co-expression modules were identified through weighted gene co-expression network analysis. Among them, 17 genes in the two modules that significantly associated with pasteurization were mainly involved in the tricarboxylic acid cycle, NF-kappa B signaling, and NOD-like receptor signaling pathways. Finally, DEGs that underwent alternative splicing in calves fed pasteurized colostrum have roles in the immune response (*SLCO4A1*, *AKR1C4*, and *MED13L*), indicative of potential roles in immune regulation. Results from multiple analytical methods used suggest that differences in calf growth between the pasteurized and unpasteurized groups may be due to differential immune activity. Our data provide new insights into the impact of pasteurization on calf immune and metabolic-related pathways through its effects on gene expression.

## 1 Introduction

The calf rearing stage is crucial and healthy calf growth has a positive impact on improving mating performance of breeding cows, as well as on overall production performance (Cummins et al., 2016). Colostrum is the milk secreted by cows for up to 2–3 days after parturition. It contains high amounts of immunoglobulins, antimicrobial peptides and growth factors. As the first meal for newborn calves, colostrum has a vital role in calf nutrition and immune defense, growth and development (Playford and Weiser, 2021). The immune status of calves after birth directly depends on the amount and quality of colostrum they consume (Silva et al., 2021). Colostrum quality significantly affects the formation of intestinal microbiota and the daily weight gain of calves, which is the highest when the colostrum density is > 1.070 g/cm^3^ (Puppel et al., 2020). In calves, adequate passive immunity increases daily gain and weaning weight, reduces the age at first mating and first calving, and increases milk production in the first lactation (Godden et al., 2019). Calves with serum IgG concentrations <10 g/L in the first 24–48 h after birth are considered to have failed to recieve passive immunization, and this increases the incidence of disease and mortality in the first week of life (Godden et al., 2019). Therefore, high quality colostrum can enhance calf immunity, health and growth performance, which is essential to maintain the economic efficiency of dairy farms.

However, colostrum is susceptible to contamination by milking equipment, storage containers, and feeding equipment, resulting in high levels of pathogenic microorganisms in colostrum such as *Mycobacterium avium* ssp. *paratuberculosis* (Sweeney, 1996), *Mycoplasma* ssp. (Stabel et al., 2004), *Escherichia coli*, and *Salmonella* (Streeter et al., 1995; Pithua et al., 2011; Slosarkova et al., 2021). These pathogens may bind immunoglobulins in the gut of the calves and affect the uptake and transport of immunoglobulins by intestinal epithelial cells, leading to passive immunization failure (Elizondo-Salazar and Heinrichs, 2009). Numerous studies have demonstrated that pasteurization can inactivate crucial pathogens such as *E. coli*, *Salmonella*, and *Listeria monocytogenes* while maintaining the quality of colostrum (Stabel et al., 2004; McMartin et al., 2006; Elizondo-Salazar and Heinrichs, 2009; Heinrichs and Elizondo-Salazar, 2009). In addition, pasteurization is effective in reducing the concentration of certain heat-stable pathogens such as *M. avium* ssp. *paratuberculosis* (Fechner et al., 2019). Pasteurized colostrum has a positive impact on healthy calf growth, and feeding pasteurized colostrum is effective in reducing the risk of upper respiratory disease and diarrhea, and mortality in calves (Armengol and Fraile, 2016; Kargar et al., 2020). Pasteurization treatment may disrupt the function of heat-unstable bioactive compounds such as immunoglobulins, proteases, and cholesterol (Sousa et al., 2014; Parron et al., 2016). However, previous study showed no significant effect of heat treatment on the composition of the colostrum metabolome and no detectable changes in the serum metabolome profile of raw colostrum compared to heat-treated colostrum fed calves (Xu W. et al., 2021).

Pasteurized colostrum is helpful for establishing passive immunity in calves. Pathogenic microorganisms are greatly reduced in pasteurized colostrum compared to unpasteurized colostrum, but changes related to gene expression and immune pathways are not known. We therefore hypothesized that feeding pasteurized colostrum to calves at birth would influence the expression of genes associated with immune responses and metabolism. In this study, RNA-seq technology and bioinformatic analysis were used to study the blood gene expression profiles of calves fed with pasteurized or unpasteurized colostrum as well as examined the effect of pasteurization treatment on calf growth traits. The study results provide better understanding of the effects of pasteurized colostrum on health status and growth performance, which may facilitate optimization of current calf feeding regimes.

## 2 Materials and methods

### 2.1 Animal and feeding management

Animal experiments were approved by the Animal Ethical and Welfare Committee of Ningxia University (approval number: NXUC20210718). The experiment was conducted on a cattle farm in Ningxia, China. Holstein female calves were selected as experimental subjects based on the following criteria: Birth weight greater than 30 kg and less than 40 kg, and calves with serum total protein less than 5.5 g/dL at 24 h were excluded (Windeyer et al., 2014). Calves with fever, diarrhea, disability, and other diseases were also excluded. A total of 32 healthy female calves (average weight 39.8 ± 1.22 kg) were randomly assigned to two groups (pasteurized and unpasteurized colostrum, 16 calves per group), and fed in individual pens where manure was removed daily to keep the pens clean and dry. Colostrum and milk were provided in buckets, which were cleaned and disinfected after each use. The trial period was 180 days in total. All tested calves were fed 6–8 L colostrum for 12 h after birth (colostrum processing for the pasteurized group is explained in Section 2.2). Subsequently, from day 2 to day 60, an average of 3 L (2.5–3.5 L) milk was fed every 12 h. From day 61 to day 180, pelleted feed and oat hay were offered *ad libitum* to all the calves (Supplementary Table S1). To reduce the stress of the change in diet, milk feeding was reduced daily for both groups (by 1.5 L per day, see Supplementary Table S2 for detailed feeding schedule) until the end of the 180-day trial. Starting on day 60, all calves were fed *ad libitum* on pellet feed and water.

### 2.2 Colostrum and milk management

The milk used was produced on the farm itself. Colostrum was from cows free of paratuberculosis and having somatic cell count less than 400,000 cells/ml, and no visible signs of inflammation or disease. Serum samples were used to test for the presence of *Mycobacterium avium* spp. *paratuberculosis* with the PARACHEK two Bovine *Mycobacterium paratuberculosis* Test Kit (Prionics AG, Schlieren-Zurich, Switzerland). Colostrum with a specific gravity of more than 1.065 was used (Fleenor and Stott, 1980; Armengol and Fraile, 2016). Colostrum was pasteurized by heating continuously at 60°C for 1 h, followed by storage at −20°C. Frozen colostrum was heated continuously at 40°C for 30 min and then fed to calves. Details of the treatment process are shown in Supplementary Figure S1. The temperature and time of pasteurization during colostrum and milk handling were strictly observed.

### 2.3 Sampling and phenotyping

For all calves in this study, traits related to growth, such as height, weight and daily weight gain were measured. The mean of each parameter for all calves in each group was taken as the phenotypic value. The data were analyzed for significance using the general linear model in SPSS 22.0, and the results were represented as “mean ± SE”, with *p* < 0.05 indicating significant differences. At the end of the test period, 5 ml whole blood was drawn from the caudal vein of each calf using disposable blood collection needles into EDTA anticoagulant tubes, and stored at −80°C until used.

### 2.4 RNA isolation and transcriptome sequencing

Total RNA was extracted from blood samples using TRIzol reagent (Invitrogen, Carlsbad, CA, United States), in accordance with the manufacturer’s instructions. RNA concentration and integrity were assessed using an Agilent 2100 BioAnalyzer (Agilent Technologies, Santa Clara, CA, United States). RNA samples with RIN ≥7 and 28S/18S values in the range 1.8–2.0 were used for sequencing. Four samples were randomly selected from each of the pasteurized and unpasteurized groups for sequencing. RNA sequencing libraries were constructed and sequenced by CapitalBio Technology Co., Ltd. (Beijing China) using the Illumina HiSeq 2500 sequencer (Illumina, San Diego, CA, United States).

### 2.5 Quality control, alignment, and identification of differentially expressed genes

The sequencing quality of the raw data was evaluated using FastQc v0.11.7 (Schmieder and Edwards, 2011), and reads were trimmed using Trimmomatic v0.39 (Bolger et al., 2014). The clean reads with average base quality greater than 20 were selected for subsequent analysis. The trimmed clean reads were aligned to the bovine reference genome (*Bos taurus* UMD3.1) using Hisat2 v2.2.1 (Kim et al., 2019) to obtain the *sam* files. The *sam* files were converted to the *bam* files using samtools v1.9 (Li et al., 2009). Meanwhile, the bam file index was generated, and expressed genes were counted using StringTie v2.1.2 (Pertea et al., 2015) to obtain the count matrix. Gene expression was quantified based on transcript per million values. Differentially expressed genes (DEGs) were detected using the R package DESeq2 v1.20 (Anders and Huber, 2010), with a differential gene screening threshold of |log2FC|≥0.585 and *p*-value corrected using the Benjamini–Hochberg false discovery rate of <0.05.

### 2.6 Functional annotation of DEGs and protein-protein interaction network analysis

Gene Ontology (GO) and Kyoto Encyclopedia of Genes and Genomes (KEGG) pathway functional annotation of DEGs was performed with DAVID v6.8 (Sherman et al., 2022). GO annotations were grouped into three broad categories, namely, cellular components, molecular function, and biological processes. Significance levels for GO terms and KEGG pathways were tested with a threshold of corrected *p* set at <0.05. Protein–protein interaction (PPI) networks were constructed using STRING v11.5 with a confidence level of >0.9 and visualized using Cytoscape v3.8.0 (Shannon et al., 2003). The Molecular Complex Detection (MCODE) program in Cytoscape was used to filter key subnetworks and nodes in the PPI network (degree cutoff = 2, node score cutoff = 0.2, k-core = 2, maximum depth = 100). The CytoHubba plugin in Cytoscape was used to detect hub genes through four methods, network topology analysis-Degree, edge percolated component (EPC), maximal clique centrality (MCC), and maximum neighborhood component (MNC). Overlapping genes screened using the four methods were selected as core genes.

### 2.7 Weighted gene Co-expression network analysis

Weighted gene co-expression network analysis (WGCNA) was used to explore the association between gene networks and sample phenotypes, as well as the core genes in the network. Gene co-expression networks were constructed using the WGCNA v1.69 package in R (Zhang and Horvath, 2005; Langfelder and Horvath, 2008). The soft threshold was determined to be seven when the correlation coefficient was 0.85. The minimum number of genes in the module was set to 300. To merge potentially parallel modules, the threshold for the height of cut was set to 0.25. To further understand the role of expressed genes in the modules most closely associated with pasteurization treatment, GO and KEGG analyses were performed using DAVID v6.8, with corrected *p* < 0.05 set as the significance threshold. The results were visualized using the ggplot2 v3.3.2 package in R (Hamilton and Ferry, 2018).

### 2.8 Gene set enrichment analysis

All expressed genes were used for gene set enrichment analysis (GSEA). Genes were sorted according to the degree of differential expression, and then a predetermined set of gene enrichments were examined at the top or bottom of this sorted table to determine whether the pathway in question was activated or repressed under experimental conditions. Analysis was conducted using the clusterProfiler v4.0.0 package in R (Yu et al., 2012), and FDR <0.05 was chosen as the selection threshold.

### 2.9 Alternative splicing analysis

Alternative splicing (AS) events in each sample were classified and counted using Asprofile (v1.0.4) software (Florea et al., 2013). rMATs v4.1.0 (Shen et al., 2014) was used to identify AS events. The frequency of AS events was quantified as the percent spliced in (PSI) based on the sorted bam files. rMATs utilizes proportions of exon-exon junction reads to calculate PSI (IncLevel) values. Significantly different AS events were assessed by ΔPSI (|IncLevel1-IncLevel2|) > 0.05 and FDR <0.01, and IncLevel1-IncLevel2 represented the difference in PSI values between the pasteurized and unpasteurized groups. By referring to the KOBAS v3.0 (Bu et al., 2021), KEGG enrichment analysis (*p*-value < 0.05) of differentially splicing genes was performed to identify significantly enriched pathways and key genes.

## 3 Results

### 3.1 Effect of pasteurized colostrum on growth traits of calves

The differences in body weight between calves in the pasteurized and unpasteurized groups in all months tested was significant (*p* < 0.05; Table 1). Calves in the pasteurized group had significantly higher body heights than calves in the unpasteurized group at 3 and 4 months of age (*p* < 0.05), but not at 1, 2, 5, and 6 months of age. With respect to daily weight gain, the pasteurized group calves had generally higher daily weight gains than the unpasteurized group; with significant differences detected at 2, 3, and 6 months of age (*p* < 0.05), but not significant at other months (*p* >0.05).

**TABLE 1:** Effect of pasteurized colostrum on body height, body weight and daily weight gain of calves.

Age (Month)	Phenotype	Group pasteurized^1^ (*n* = 16)	Group unpasteurized (*n* = 16)	*p*-value
1 month	Body weight (kg)^2^	71.27 ± 2.34^b^	66.25 ± 1.88^a^	0.02
	Body height (cm)	89.00 ± 0.76	87.69 ± 1.96	0.21
	Daily weight gain (kg)	1.09 ± 0.04	0.83 ± 0.03	0.16
2 months	Body weight (kg)	96.53 ± 2.12^B^	88.25 ± 2.87^A^	0.00
	Body height (cm)	95.67 ± 0.76	94.44 ± 1.31	0.17
	Daily weight gain (kg)	0.84 ± 0.03^B^	0.74 ± 0.05^A^	0.00
3 months	Body weight (kg)	134.33 ± 1.71^B^	124.44 ± 2.98^A^	0.00
	Body height (cm)	105.00 ± 0.78^b^	102.38 ± 1.18^a^	0.03
	Daily weight gain (kg)	1.27 ± 0.04^B^	1.19 ± 0.03^A^	0.00
4 months	Body weight (kg)	163.33 ± 2.11^B^	155.25 ± 3.14^A^	0.00
	Body height (cm)	111.07 ± 0.85^b^	108.50 ± 1.33^a^	0.03
	Daily weight gain (kg)	0.97 ± 0.02	1.03 ± 0.03	0.71
5 months	Body weight (kg)	200.07 ± 3.06^B^	191.13 ± 2.86^A^	0.00
	Body height (cm)	115.53 ± 0.77	113.88 ± 0.61	0.06
	Daily weight gain (kg)	1.23 ± 0.05	1.20 ± 0.02	0.91
6 months	Body weight (kg)	228.87 ± 2.84^b^	225.38 ± 3.13^a^	0.04
	Body height (cm)	120.60 ± 1.08	119.38 ± 1.15	0.15
	Daily weight gain (kg)	0.96 ± 0.03^b^	1.14 ± 0.04^a^	0.02

1 Values within a row with different superscript letters are significantly different. Lowercase letters indicate significant differences (p < 0.05) and uppercase letters indicate highly significant differences (p < 0.01).

2 Reported values reflect mean ± SE.

### 3.2 Sequencing data mapping statistics

Transcriptome sequencing was performed on eight whole blood samples. An average of 53.86 million raw reads were generated per sample. After filtration, the clean reads ranged from 48,192,466 to 52,812,286 (>96.21%). The Q20 values ranged from 97.08% to 97.52%. On average, 94.55% of the reads were mapped to the reference genome (Table 2).

**TABLE 2:** Quality analysis of sequencing data.

Sample		Raw reads	Q20^a^ (%)	Clean reads	Clean reads ratio (%)	Mapping reads	Mapping ratio (%)
unpasteurized	15087	54282542	97.39	52324844	96.39	49561457	94.72
	15089	54531096	97.52	52605846	96.47	49530386	94.15
	15198	49893692	97.25	48192466	96.59	45815093	95.07
	15201	54044380	97.33	52108504	96.42	49190649	94.40
pasteurized	15084	54653568	97.10	52534432	96.12	49630238	94.47
	15123	54759418	97.50	52812286	96.44	49862821	94.42
	15124	54089090	97.45	51879160	95.91	49197132	94.83
	15189	54653568	97.08	52087152	95.30	49137874	94.34

a Probability of base being misidentified is 1%.

### 3.3 DEGs and functional enrichment analysis

**FIGURE 1:**
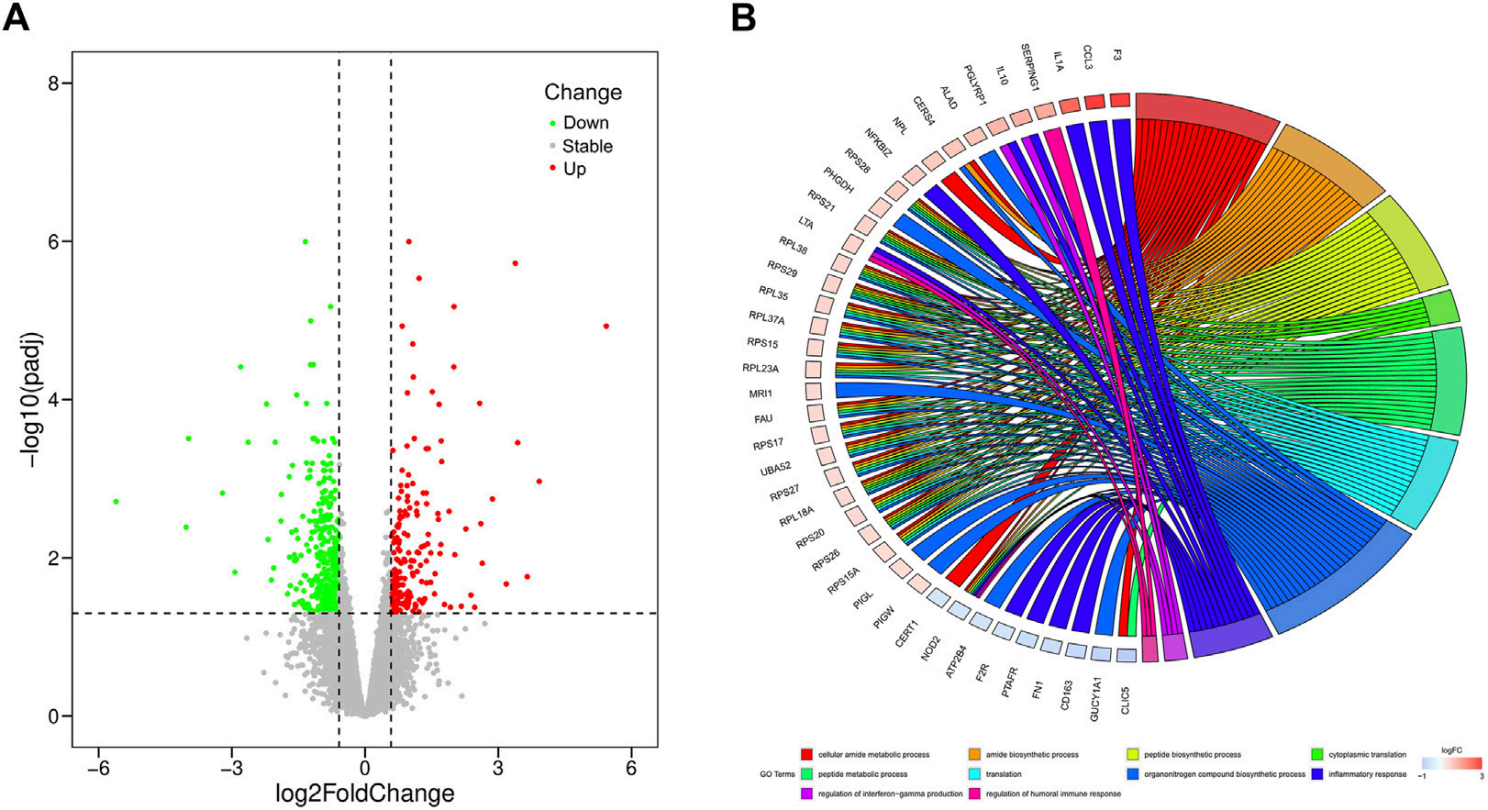
Volcano and circos plots of differentially expressed genes. **(A)** Volcano plot for expressed genes, it represents the -log10 (*p*-value) (*y*-axis) of genome-wide genes in relation to their respective log2 (fold change) (*x*-axis). Red dots represent up-regulated genes, green dots represent down-regulated genes, and gray dots represent genes with no significant difference in expression (|log2FC|≥0.585 and FDR<0.05). **(B)** Circos plot for functional enrichment of DEGs. The color gradient from blue to red on the left side of the graph represents the change of genes from down-regulation to up-regulation, and the terms on the right side are represented by different colors.

In our study, a total of 20,679 expressed genes were detected and 630 DEGs were obtained. Of these, 235 DEGs were up-regulated and 395 were down-regulated in the pasteurized group (compared to the unpasteurized group) (Figure 1A). The top 10 upregulated and downregulated genes in the pasteurized groups are listed in Table 3. Among these genes, chemokine ligand 3 (*CCL3*), CXC motif chemokine ligand 2 (*CXCL2*), interleukin one alpha (*IL1A*), multiple EGF like domains 11 (*MEGF11*), and pentraxin 3 (*PTX3*) are associated with growth as well as the immune response in calves.

**TABLE 3:** The top 10 up- or down-regulated genes identified in pasteurized group.

Symbol^a^	Basemean	Log2FC^b^	*p-*value	*P*adj^c^	Change^d^
*F3*	67.44	3.38	4.36E-10	1.89E-06	Up
*CCL3*	1574.52	3.18	6.38E-04	2.14E-02	Up
*WC1-10*	219.92	2.64	2.54E-04	1.17E-02	Up
*RND1*	19.85	2.61	3.85E-05	3.70E-03	Up
*EDN1*	104.65	2.58	1.63E-07	1.12E-04	Up
*IL1A*	197.42	2.47	2.56E-03	4.22E-02	Up
*DEFB4A*	33.96	2.38	1.21E-03	2.96E-02	Up
*CXCL2*	2605.93	2.27	5.03E-05	4.30E-03	Up
*TFPI2*	22.76	2.17	2.44E-03	4.08E-02	Up
*DEFB10*	85.33	2.00	3.08E-09	6.69E-06	Up
*MEGF11*	107.19	−5.61	1.28E-05	1.94E-03	Down
*PTX3*	79.68	−2.93	3.63E-04	1.52E-02	Down
*MIR223*	70.65	−2.63	8.76E-07	3.46E-04	Down
*SLC43A3*	63.58	−2.22	1.92E-07	1.14E-04	Down
*MIR27A*	22.46	−2.18	8.05E-05	5.86E-03	Down
*CPXM2*	191.37	−2.02	9.03E-07	3.46E-04	Down
*EXTL1*	32.78	−1.89	3.43E-05	3.42E-03	Down
*CTTNBP2NL*	94.50	−1.88	9.24E-06	1.58E-03	Down
*SLC4A8*	20.46	−1.74	1.10E-03	2.86E-02	Down
*CDH20*	61.57	−1.71	4.04E-06	9.41E-04	Down

a Gene symbol in NCBI.

b Logarithm of fold change with a base of 2.

c Adjust *p*-value.

d Genes with downregulation or upregulation in pasteurized (n = 4) compared to unpasteurized group (*n* = 4).

To investigate the relationship between DEGs and pasteurization, we performed GO enrichment analysis (Figure 1B). These DEGs were mainly enriched in protein anabolic pathways such as cytoplasmic translation, peptide biosynthesis, and peptide metabolism. They were also involved in pathways related to the immune response, such as the humoral immune response, negative regulation of the immune effector process, and the regulation of IL8 production. Some identified DEGs are among genes of signaling pathways involved in cellular substance metabolism, such as cellular amide metabolic processes, amide biosynthetic processes, and calcium ion transmembrane import into the cytosol. KEGG analysis revealed that 13 pathways were significantly enriched between the pasteurized and unpasteurized groups (Supplementary Figure S2) (corrected *p* < 0.05). Most enriched pathways were related to immune responses, including the cytokine–cytokine receptor interactions, tumor necrosis factor (TNF) signaling pathway, and chemokine signaling pathway. Some pathways were related to metabolism, including ribosome, thermogenic, and gap junction pathways. From these results we inferred that growth and developmental differences between pasteurized and unpasteurized groups may be attributed to protein function, immune response, and metabolic efficiency.

### 3.4 Protein-protein interaction network analysis

**FIGURE 2:**
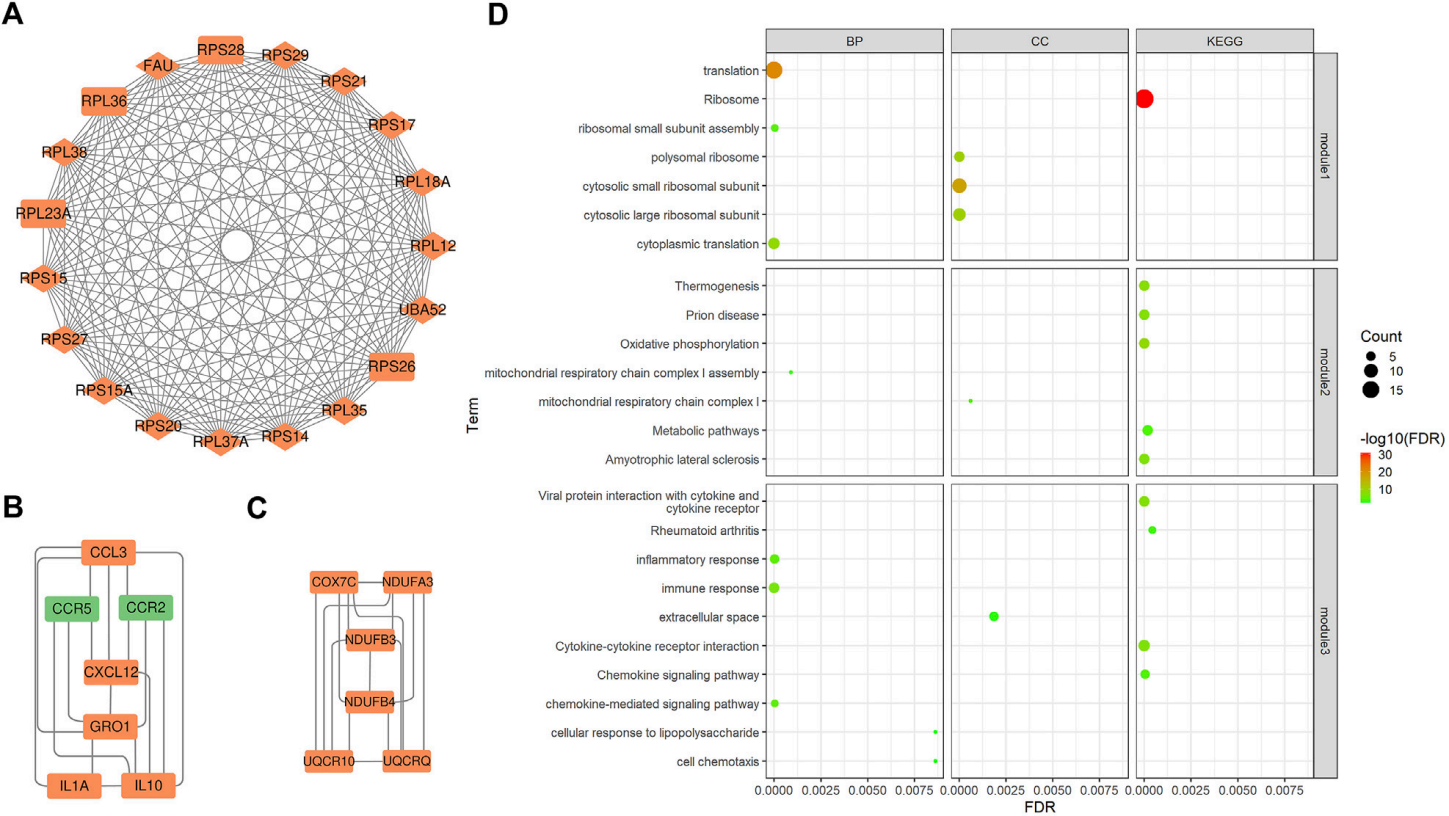
Key subnetworks from PPI networks and their enrichment analysis. **(A)**, **(B)**, and **(C)** Three key subnetworks. The red and green nodes represent the up- and down-regulated genes in the shapes of “diamond” and “rectangle”, represent core genes and DEGs. **(D)**The bubble plots of functional enrichment analysis of the three key subnetworks. The size of the dots represents the number of genes in the pathway and the color represents the pathway significance.

PPI is mainly used to study the interaction network between proteins, which helps to identify core regulatory genes. PPI analysis led to the construction of a PPI network containing 158 nodes and 443 edges. The top 15 DEGs (Supplementary Table S3) were identified through four methods (Degree, EPC, MCC, and MNC) in the CytoHubba plugin and overlapping DEGs were considered core genes (*UBA52*, *RPS28*, *RPS14*, *FAU*, *RPS15*, *RPS17*, *RPS29*, *RPS20*, *RPL18A*, *RPS21*, *RPL38*, *RPL37A*, *RPL35*, *RPL36*, and *RPS15A*). Most genes encoded ribosomal proteins, which play key roles in ribosome assembly and function. They also regulate protein synthesis and metabolic activities in response to cell growth and proliferation, and therefore play key roles in calf growth. In addition, three key subnetworks (Figures 2A–C) were identified through MCODE analysis: subnetwork 1 (Score = 18.889) had 19 nodes and 170 edges, subnetwork 2 (Score = 6.000) had six nodes and 15 edges, and subnetwork 3 (Score = 5.667) had seven nodes and 17 edges. Our analysis revealed that the core genes were mainly present in subnetwork one.

To understand the biological functions involved in the key subnetworks, a functional enrichment analysis (corrected *p* < 0.05) was performed (Figure 2D). According to the results, genes in subnetwork one were significantly enriched in ribosome-related pathways, such as polysomes ribosomes, ribosomes, and small ribosomal subunits, as well as in translation-related pathways including, translation, cytoplasmic translation, and RNA binding. Genes in subnetwork two were significantly enriched in electron transport-related pathways, such as mitochondrial respiratory chain complex I and oxidative phosphorylation pathways. Genes in subnetwork three were significantly enriched in pathways related to immune responses, such as the chemokine signaling, the MAPK cascade and the NF-kappa B signaling pathways.

### 3.5 Weighted gene Co-expression network analysis

**FIGURE 3:**
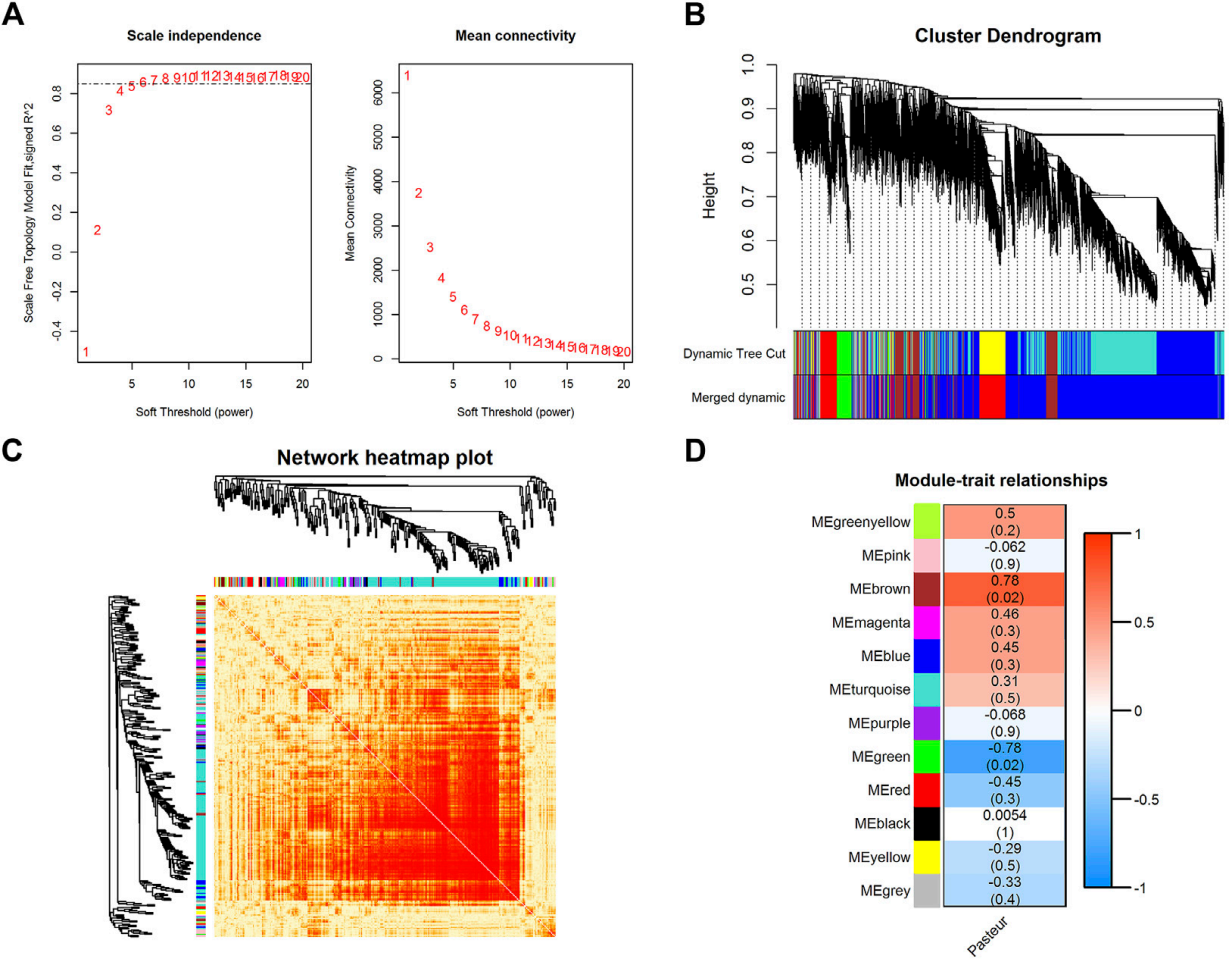
WGCNA of all expressed genes. **(A)** The analysis of the scale-free fit index and mean connectivity for diverse soft-thresholding powers. **(B)**Hierarchical clustering dendrograms showing 12 modules of co-expressed genes. Each branch in the clustering tree represents a gene, while co-expression modules were constructed in different colors. **(C)** Network heatmap in the co-expression modules (the yellow color scale indicates the degree of overlap between functional modules). **(D)** Module-trait association heatmap where each row corresponds to a module eigengene and the columns correspond to a trait. Each cell contains the correlation and *p*-value for the corresponding differentiation stages. The scale bar indicates the color coding for the correlations, with blue to red indicating low to high correlations, respectively.

WGCNA is a powerful tool for finding clusters (modules) of highly correlated genes (Xing et al., 2021; Yang et al., 2021; Yang et al., 2022). The soft threshold power was chosen to be seven when 0.85 was used as the correlation coefficient threshold (Figure 3A). Twelve co-expression modules were constructed through WGCNA analysis (Figure 3B). The major modules were the turquoise module (7287 genes), blue module (1431 genes), brown module (817 genes), yellow module (663 genes), and green module (573 genes). These modules were independent of each other (Figure 3C).

After the gene co-expression modules were obtained, the modules were correlated with phenotype. The results showed that the brown module (*r* = 0.78, *p* = 0.02) and the green module (*r* = −0.78, *p* = 0.02) were significantly correlated with the phenotype (Figure 3D; Supplementary Figure S3), containing 817 and 573 genes, respectively. Among these two modules, *PFKFB3, CALHM2, FBXO8, UTP15, CRK, METTL9, TMEM80, FBXW7, CLEC4A, NUDT4, TCN2, ROPN1L, GRAMD1A, AIMP2, CDKN1A, TDRD6,* and *MST1* had a high gene significance with phenotype (Supplementary Table S4) (|Gene significance| > 0.8), and were considered as key genes (Supplementary Figures S4, S5). KEGG enrichment analysis of key genes (corrected *p* < 0.05) (Supplementary Figures S6, S7) in both modules revealed that both modules were significantly enriched in pathways related to mitochondrial function, such as mitochondrial matrix, oxidative phosphorylation, and reactive oxygen species production pathways. They were also closely related to substance and energy metabolism, such as carbon metabolism, fatty acid metabolism, the biosynthesis of amino acids, and the tricarboxylic acid cycle. In addition, they were involved in immune-related pathways such as the IL17 signaling pathway, the TNF signaling pathway, the NF-kappa B signaling pathway, and the NOD-like receptor (NLR) signaling pathway. These results suggest that key genes in both modules were involved in substance and energy metabolism, and immune-related pathways, which may be closely related to the effects of colostrum pasteurization treatment on calf vital activity.

### 3.6 Gene set enrichment analysis

All 10 gene sets were significantly enriched in the pasteurized group (Table 4; Supplementary Figure S8). These gene sets are mainly involved in cellular substance and energy metabolism pathways, such as oxidative phosphorylation, proteasome metabolism, and arachidonic acid metabolism pathways. They are also involved in immune-related pathways, such as the cytosolic DNA sensing pathway, the NLR signaling pathway, and the TOLL-like receptor signaling pathway. These results have shown that colostrum pasteurization affected late growth in calves, probably mainly through modulation of immune and metabolic pathways.

**TABLE 4:** List of all gene sets with an FDR <0.05.

KEGG_set^a^	SetSize^b^	NES^c^	*p-*value^d^	*p*.adjust^e^	Qvalues^f^	Higher expression
KEGG_RIBOSOME	84	2.612692311	1.00E-10	1.80E-08	1.66E-08	pasteurized
KEGG_OXIDATIVE_PHOSPHORYLATION	112	1.996094851	9.31E-07	8.38E-05	7.74E-05	pasteurized
KEGG_PARKINSONS_DISEASE	110	1.848321432	2.32E-05	0.001394393	0.001288386	pasteurized
KEGG_CYTOSOLIC_DNA_SENSING_PATHWAY	40	1.953656946	9.22E-05	0.004148699	0.003833301	pasteurized
KEGG_NOD_LIKE_RECEPTOR_SIGNALING_PATHWAY	54	1.846558802	0.000328417	0.01072163	0.009906535	pasteurized
KEGG_PROTEASOME	43	1.811537622	0.000357388	0.01072163	0.009906535	pasteurized
KEGG_TOLL_LIKE_RECEPTOR_SIGNALING_PATHWAY	88	1.680005241	0.001060941	0.025787898	0.023827414	pasteurized
KEGG_SYSTEMIC_LUPUS_ERYTHEMATOSUS	89	1.661668017	0.001146129	0.025787898	0.023827414	pasteurized
KEGG_ALZHEIMERS_DISEASE	146	1.556932809	0.001394052	0.027881043	0.025761431	pasteurized
KEGG_ARACHIDONIC_ACID_METABOLISM	38	1.711590154	0.002505201	0.045093626	0.041665456	pasteurized

a Name of the gene sets.

b Total number of genes under gene sets.

c Normalized enrichment score.

d *p*-value of the statistical test.

e Corrected *p*-value.

f False discovery rate.

### 3.7 Alternative splicing analysis

In an mRNA precursor, different exons usually have different AS patterns. There are five common types of AS patterns, namely skipped exon (SE), alternative 5′splice site (A5SS), alternative 3′splice site (A3SS), mutually exclusive exons (MXE), and retained intron (RI) patterns. In the blood of calves fed pasteurized colostrum, the incidence of A5SS and SE patterns were the highest, followed by RI, A3SS, and MXE patterns (Table 5). rMATS detected differential AS events, including 136 genes of the SE type, 10 genes of the MXE type, and one gene of the SE type (Supplementary Table S5).

**TABLE 5:** Alternative splicing events statistics of pasteurized and unpasteurized groups.

Sample	Group	A5SS^a^	A3SS^b^	MXE^c^	SE^d^	RI^e^
15201	pasteurized	7138	4662	4490	9228	5944
15087	pasteurized	6884	4059	3917	7809	5518
15089	pasteurized	7034	4381	4060	9004	4684
15198	pasteurized	7638	4454	5149	8899	9011
Total	—	28694	17556	17616	34940	25157
15124	unpasteurized	6478	4295	3549	7442	3887
15189	unpasteurized	6690	4199	3957	7820	5286
15084	unpasteurized	6983	4325	3905	8562	4582
15123	unpasteurized	7179	4371	4096	8304	5695
Total	—	27330	17190	15507	32128	19450

a A5SS, Alternative 5′splice site.

b A3SS, Alternative 3′splice site.

c MXE, mutually exclusive exons.

d SE, skipped exon.

e RI, retained intron.

**FIGURE 4:**
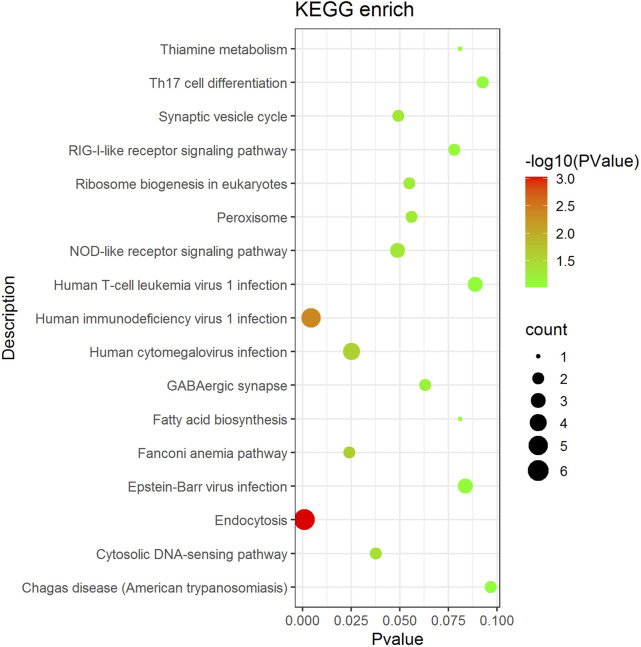
Main pathway enrichment of AS genes. The dot size represents the number of genes in the pathway, the color represents the pathway significance and the vertical coordinate represents the KEGG pathway.

KEGG enrichment analysis was performed on genes associated with differential AS events (Figure 4). The enriched pathways, namely, the RIG-I-like receptor signaling pathway, the cytosolic DNA-sensing pathway, the NLR signaling pathway, endocytosis, and the Th17 cell differentiation pathway, which influence the immune response activity and are associated with substance and energy metabolism-related pathways, such as ribosome biogenesis in eukaryotes, peroxisome, thiamine metabolism, and fatty acid biosynthesis.

## 4 Discussion

Colostrum quality can directly affect the growth and health of calves (Armengol and Fraile, 2020), and consequently farm sustainability. In the last few years, studies have used different histological methods to identify the effects of different milk qualities on animal growth. However, the results obtained from studies based on different animal species and tissue types were highly variable (Crisa et al., 2016; Lu et al., 2016; Xu W. et al., 2021; Keel et al., 2021). Colostrum absorption has an impact on the serum composition of newborn calves; first, colostrum contains a high amount of immunoglobulins and newborn calves must acquire passive immunity from colostrum (Blum, 2006; Mason et al., 2022). Second, colostrum is enriched with nutrient-active substances, including lactic acid, amino acids, and glycerol. Study has shown that following the intake of colostrum, calves have higher serum concentrations of glutamate, histidine, asparagine, thymidine, palmitic, and linolenic acids; these are metabolites associated with the biosynthesis of unsaturated fatty acids as well as nitrogen and galactose metabolism (Qi et al., 2018). In addition, colostrum contains other potentially bioactive substances, such as hormones and growth factors. These bioactive substances stimulate and promote intestinal epithelium growth in newborns and improve the function and absorption capacity of the gastrointestinal tract (Hammon et al., 2013; Fischer et al., 2018). Therefore, early intake of pasteurized colostrum is essential for the health, as well as the growth of newborn calves. Considering the above reasons, blood was selected as the sample for RNA-seq in this study to research the effect of gene expression changes caused by pasteurized colostrum on the growth traits in calves.

As expected, no significant difference was observed between the two groups of calves in terms of newborn weight, however the test calves fed pasteurized colostrum had significantly higher body weights, in all months tested, than the control group (unpasteurized colostrum). The pasteurized group had significantly higher body heights at 3 and 4 months of age than the unpasteurized group. Significant differences in daily weight gain were observed between the pasteurized and unpasteurized groups at 2, 3 and 6 months of age, indicating that pasteurized colostrum is crucial for the growth and development of calves. Study has proven that pasteurized colostrum not only reduces pre-weaning calf morbidity and diarrhea but also increases body weight and daily weight gain (Kargar et al., 2020). The results of the present experiment are generally consistent with those of the previous study. These results highlight that pasteurized colostrum has positive effects on the growth and development of calves.

In this study, transcriptome sequencing was used to screen the effect of pasteurized *versus* unpasteurized colostrum on blood-expressed genes in calves. In total, 630 DEGs (235 up-regulated and 395 down-regulated genes) were detected, and GO and KEGG enrichment analyses showed that DEGs were mainly enriched in biological processes such as the immune response, substance and energy metabolism. The exposure of calves to pathogenic bacteria through colostrum may lead to disease (Godden et al., 2019), so the associated immune response may be related to pathogenic bacteria in colostrum. Furthermore, by PPI analysis, we identified some key genes, such as *IL10*, *NOD2*, *CCL3*, and *COX7C*. Although these DEGs were not validated by qRT-PCR in this study, they have been reported to be associated with organismal immunity. IL10 may be an important immune response regulator in the central nervous system, mainly involved in chronic infections and neurodegenerative processes, and thus may affect internal environment homeostasis and behavior (Saraiva and O'Garra, 2010; Kwilasz et al., 2015). In humans and mice, *IL10* was found to have a crucial role in the prevention of inflammatory bowel disease (IBD) (Kuhn et al., 1993). *IL10* is also involved in immune regulation in newborns (Bullens et al., 2012; Raedler et al., 2013), suggesting that colostrum containing high amounts of immunoglobulins may be involved in the IL10-related cytokine–cytokine receptor interaction pathway in newborn calves. *NOD2* and IBD are closely related, *NOD2* has a host defense function in the gut mucosa and inhibits IBD by down-regulating *RIP2* expression (Watanabe et al., 2019). *NOD2* regulates NF-κB and MAPK pathways through *RIP2*, which in turn induces gene expression of pro-inflammatory cytokines and mediators (McDaniel et al., 2016; Zaidi and Wine, 2018). *CCL3* is a chemokine involved in various inflammatory responses through chemokine receptors (namely CCR1 and CCR5), and has a key role in several human diseases such as cancer and HIV infection (Jones et al., 2011; Kanzler et al., 2012; Kanegasaki et al., 2014). *COX7C* is a subunit of cytochrome c oxidase and is responsible for electron transfer from cytochrome c to oxygen (Hofmann et al., 1998). In addition, studies have shown that *COX7C* is a key regulator in the physiological processes of numerous diseases, where upregulation of this gene is associated with the development of many cancer tumors (Wang et al., 2017; Wang et al., 2022) and its downregulation is a feature of many chronic kidney diseases (Zaza et al., 2013; Wu et al., 2020).

WGCNA and GSEA analyses revealed that most pathways enriched were related to the immune response and metabolism, in which the NLR signaling, fatty acid metabolism and cytosolic DNA sensing pathways were mostly observed. The NLR signaling pathway has an important role in the regulation of innate immune antiviral effects. Study has shown that NLRs are closely linked to IBD and the role of *NOD2* recognition of intestinal microbiota, which is vital for maintaining intestinal immune cell homeostasis (Jiang et al., 2013). *NOD2* signaling promotes hyper-reactive macrophages and colitis in *IL10*-deficient mice, and *NOD2* deficiency in IL10^−/−^ mice leads to a significant improvement in chronic colitis (Jamontt et al., 2013). Fatty acids, major energy substances in mammals, act as signaling molecules involved in energy metabolism in humans and animals. The cytosolic DNA sensing pathway is an evolutionarily conserved mechanism that initiates a rapid innate immune response to microbial pathogens (Kwon and Bakhoum, 2020). This pathway may be related to the immune response induced by pathogenic microorganisms in colostrum.

Alternative splicing (AS) is widely present in eukaryotic organisms and specific splicing isoforms are produced in different tissues or developmental stages. Thus, gene expression changes in AS events are associated with organismal life activities and diseases. In this study, feeding calves colostrum with or without pasteurization revealed that some genes involved in signaling pathways in calves underwent AS, such as genes involved in endocytosis (*BOLA-NC1*, *AP2M1*, *SPG21*, *GIT2*, and *SMAD2*) and the NLR signaling pathway (*RIPK1*, *TMEM173*, and *MEFV*). Major histocompatibility complex class I (MHC-I) molecules are cell surface glycoproteins that play a key role in triggering immune responses (Shi et al., 2018). Of them, BOLA-NC1 is one of the non-classical (MHC-Ib) isoforms. BOLA-NC1 is a critical immunomodulatory molecule that interacts with inhibitory receptors expressed by natural killer cells, T lymphocytes, and antigen-presenting cells, thereby inhibiting these immune cells (Parasar et al., 2016; Geng and Raghavan, 2021). *RIPK1* is an upstream regulator that controls cell survival and inflammatory signaling as well as multiple cell death pathways, including apoptosis and necroptosis (Xu D. C. et al., 2021). *RIPK1* deficiency leads to the accumulation of aspartate in starved cells and tissues, which in turn inhibits AMPK signaling pathway and cellular autophagy, and increases tricarboxylic acid (TCA) activity and production of the energy metabolite ATP, thereby resulting in impaired regulation of starvation stress under starvation conditions (Mei et al., 2021). Pathogen DNA from infection or DNA from damaged cells stimulates STING (stimulator of interferon genes)-dependent type I interferon production and promotes inflammation (Ishikawa and Barber, 2008). Furthermore, the *TMEM173* gene encodes the STING protein, a key player in host defense against pathogens (Patel and Jin, 2019; Zhang et al., 2020).

A comparison of differentially spliced genes and DEGs revealed five overlapping genes, namely *SLCO4A1, AKR1C4, MED13L*, *SENP5*, and *C16orf72*. Among them, *AKR1C4* play roles in immune and metabolic processes (Onsurathum et al., 2018). *AKR1C4* is a liver-specific gene that regulates vital metabolism pathways, such as steroid hormone metabolism, bile acid synthesis and xenobiotic metabolism (Rižner and Penning, 2014). *SLCO4A1*, a potential biomarker for various cancers, is involved in neutrophil-mediated immune responses (Ban et al., 2017; Wang et al., 2021). *MED13L* play key roles in gene transcription and in the inflammatory response through participation in the Wnt signaling pathway. *MED13L* can also participate in the inflammatory response and positively regulate the secretion of pro-inflammatory factors by regulating and activating transcription factor 4 expression (Iwasaki et al., 2014; Asadollahi et al., 2017). In addition, *MED13L* was reported to be involved in FGF and Rb/E2F-related pathways, thus playing a crucial role in the regulation of immune responses (Angus and Nevins, 2012; Utami et al., 2014). Genes that undergo AS are mainly involved in intercellular inflammatory responses, signaling, and metabolic activities. However, the mechanisms underlying the role of AS genes in the immune response to pathogenic bacteria in colostrum are not well studied and need to be explored further.

Our results exemplify the intricate relationship between metabolism and immune, especially in DEGs functional enrichment. The immune system requires large amounts of energy to function, and immune cells can reprogram their metabolism to meet these energy demands. A common example of metabolic reprogramming is the coupling of the mitochondrial TCA cycle to oxidative phosphorylation (OXPHOS), which is an efficient way of energy production (Chou et al., 2022). During *Brucella* infection, *TMEM173* activation polarizes M2 macrophages to M1 macrophages, inhibits prolyl hydroxylase (PHD) activity, and increases mitochondrial ROS, thereby stabilizing hypoxia-inducible factor-1α (HIF-1α). This leads to a decrease in OXPHOS and an increase in glycolysis, thereby altering cellular immunometabolism (Downey et al., 2014; Gomes et al., 2021). The NLR signaling pathway recognizes specific pathogen-associated molecules in the cytoplasm or host-derived injury signals and activates inflammatory responses, and the *NLRP3* is a member of the NLR family, NLPR3 activation could result in the production of pro-inflammatory cytokines (Saxena and Yeretssian, 2014; Kim et al., 2016). *Salmonella* infection causes disruption of glycolysis, leading to a decrease in NAD^+^/NADH, which activates *NLRP3* inflammasome (Palsson-McDermott et al., 2015; Sanman et al., 2016). *NLRP3* inflammasome promotes glycolysis while reducing OXPHOS, resulting in the accumulation of the TCA cycle intermediate succinate, succinate inhibits PHD and promotes HIF-1α stabilization, leading to the upregulation of glycolytic enzymes (Selak et al., 2005; Tannahill et al., 2013; Parsamanesh et al., 2019). Thus, the activation of the *NLRP3* inflammasome creates a metabolic cycle. During the metabolic activity of calves, pathogenic microorganisms interfere with relevant metabolic circuits, leading to the accumulation of specific endogenous substances that induce an inflammatory response.

Although we identified DEGs and pathways underlying the effects of pasteurized colostrum on calf growth, our study has some limitations. First, the sample size used for RNA-seq was too small and needs to be further expanded to improve our study reproducibility. Second, we used blood as the study sample, which is a non-invasive and cost-saving method; however, the results of the assay may be compromised compared to more invasive tissue samples. Our future work will focus on the validation of the DEGs and pathways in additional animals.

## 5 Conclusion

The present study identified DEGs in the blood of calves fed with pasteurized and unpasteurized colostrum. The DEGs were mainly enriched in immune responses and energy metabolism pathways, which have key roles in regulating calf growth. Our study identifies the molecular mechanisms involved in the beneficial effects of pasteurized colostrum on calf growth, which will help optimize calf colostrum management practices.

## Data Availability

The data presented in the study are deposited in the NCBI repository, accession number PRJNA893578.
