# Supplementary material for: Blood transcriptome reveals immune and metabolic-related genes involved in growth of pasteurized colostrum-fed calves
Source: Front Genet. 2023 Feb 6;14:1075950. doi: 10.3389/fgene.2023.1075950 (PMC9939824; doi:10.3389/fgene.2023.1075950)
Supplement: Supplementary file 1 [file DataSheet1.PDF]

Supplementary Material

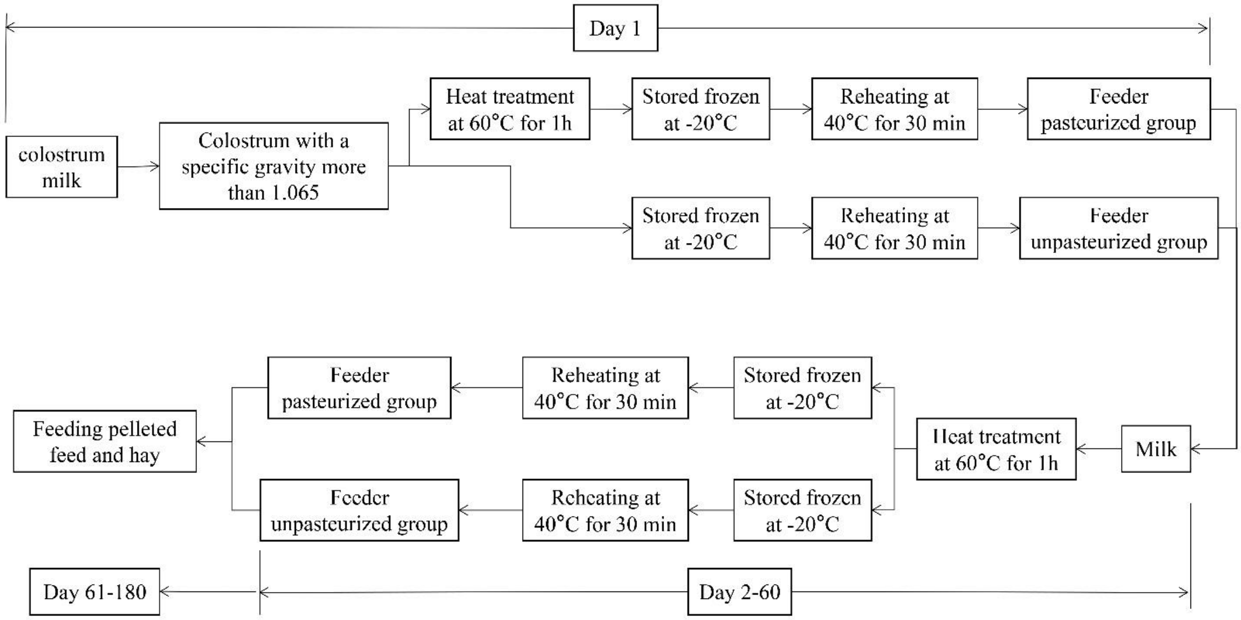

Supplementary Figure S1

Processing of colostrum and milk in the pasteurized colostrum group (n=16) and unpasteurized colostrum group (n=16).

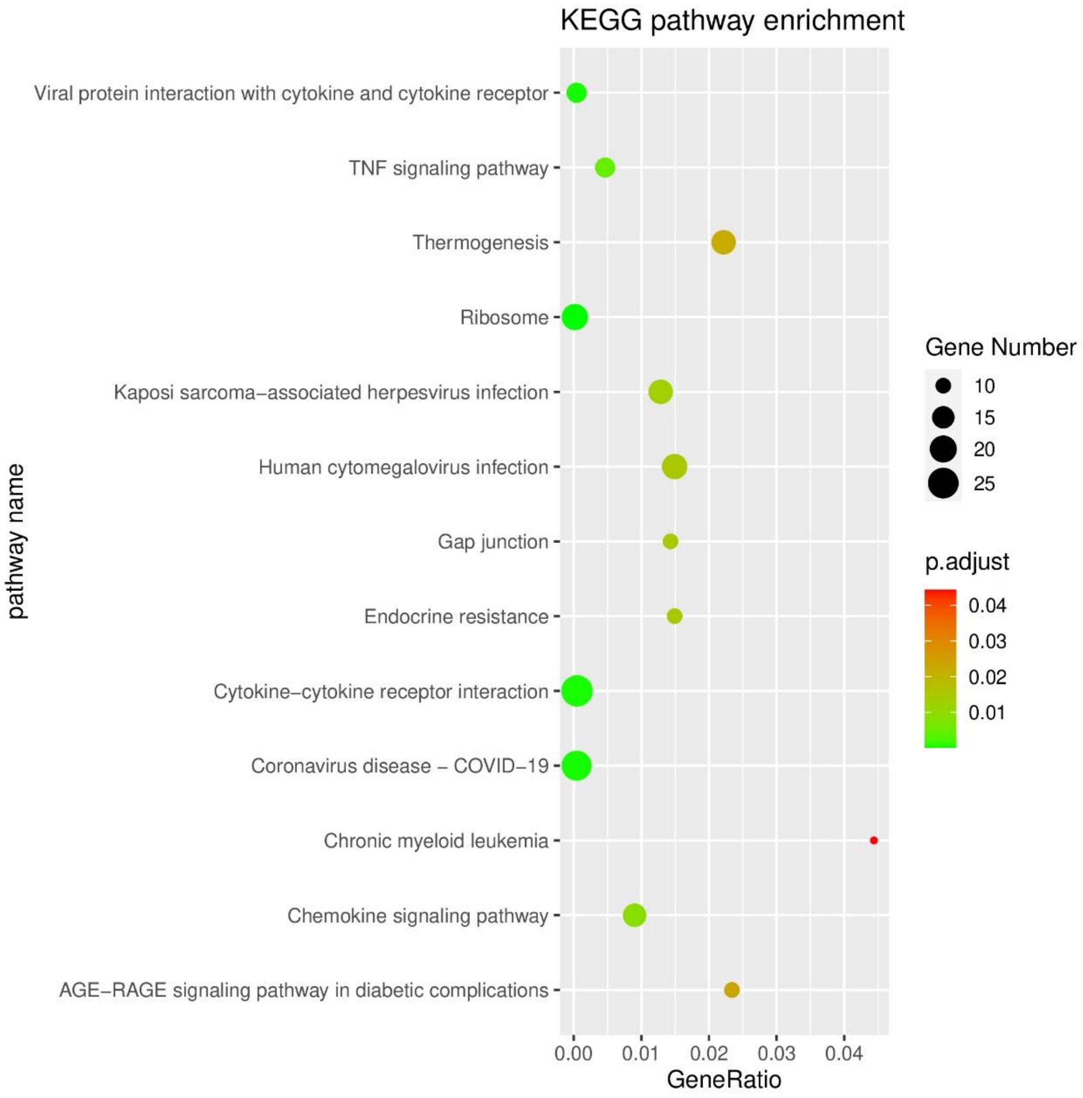

Supplementary Figure S2.

KEGG enrichment analysis of differentially expressed genes. The dot size represents the number of genes in the pathway and the color represents the pathway significance.

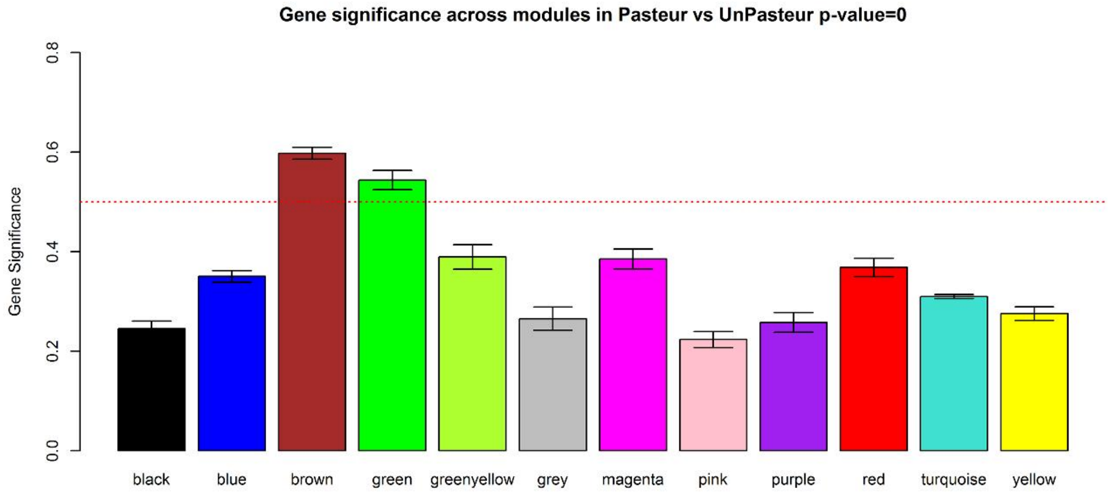

Supplementary Figure S3.

shows the absolute correlation between the genes in each module and pasteurization treatment.

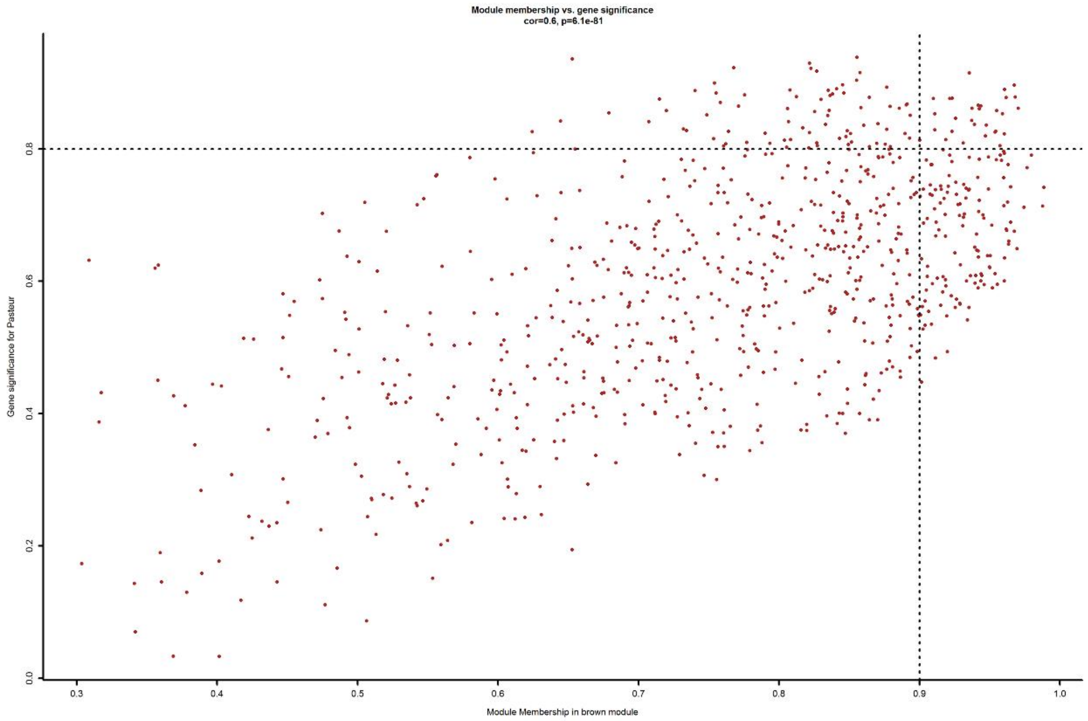

Supplementary Figure S4.

A scatterplot of Gene Significance (|GS| >0.8) for pasteurization vs. Module Membership (|MM| >0.8) in the brown module.

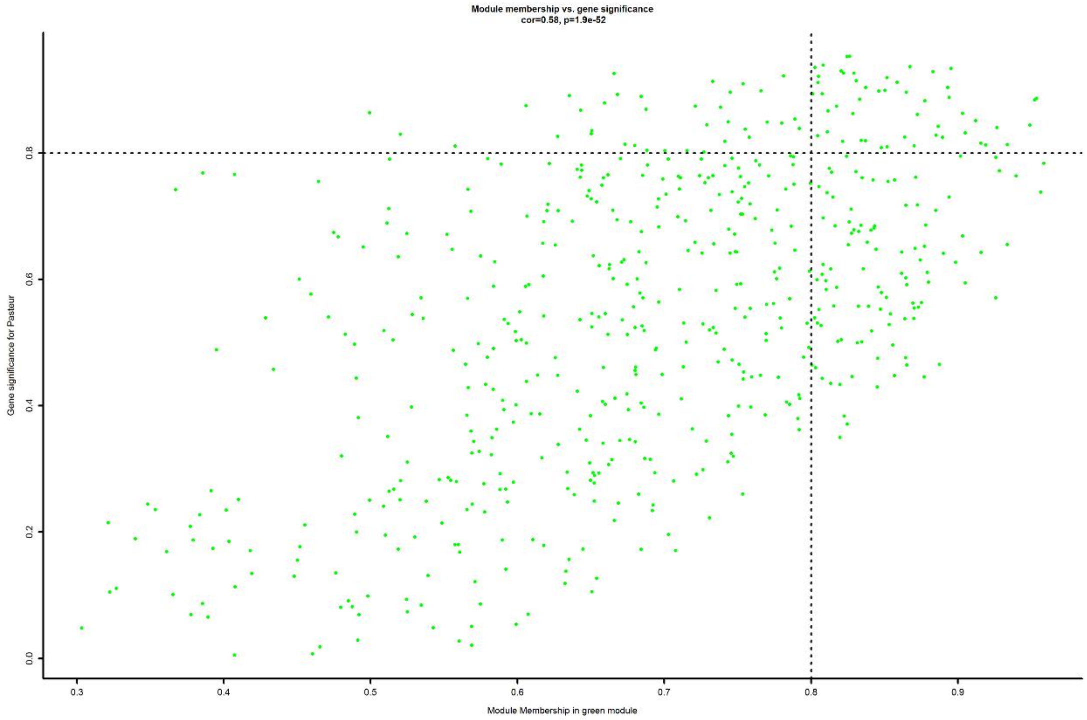

Supplementary Figure S5.

A scatterplot of Gene Significance (|GS| >0.8) for pasteurization vs. Module Membership (|MM| >0.8) in the green module.

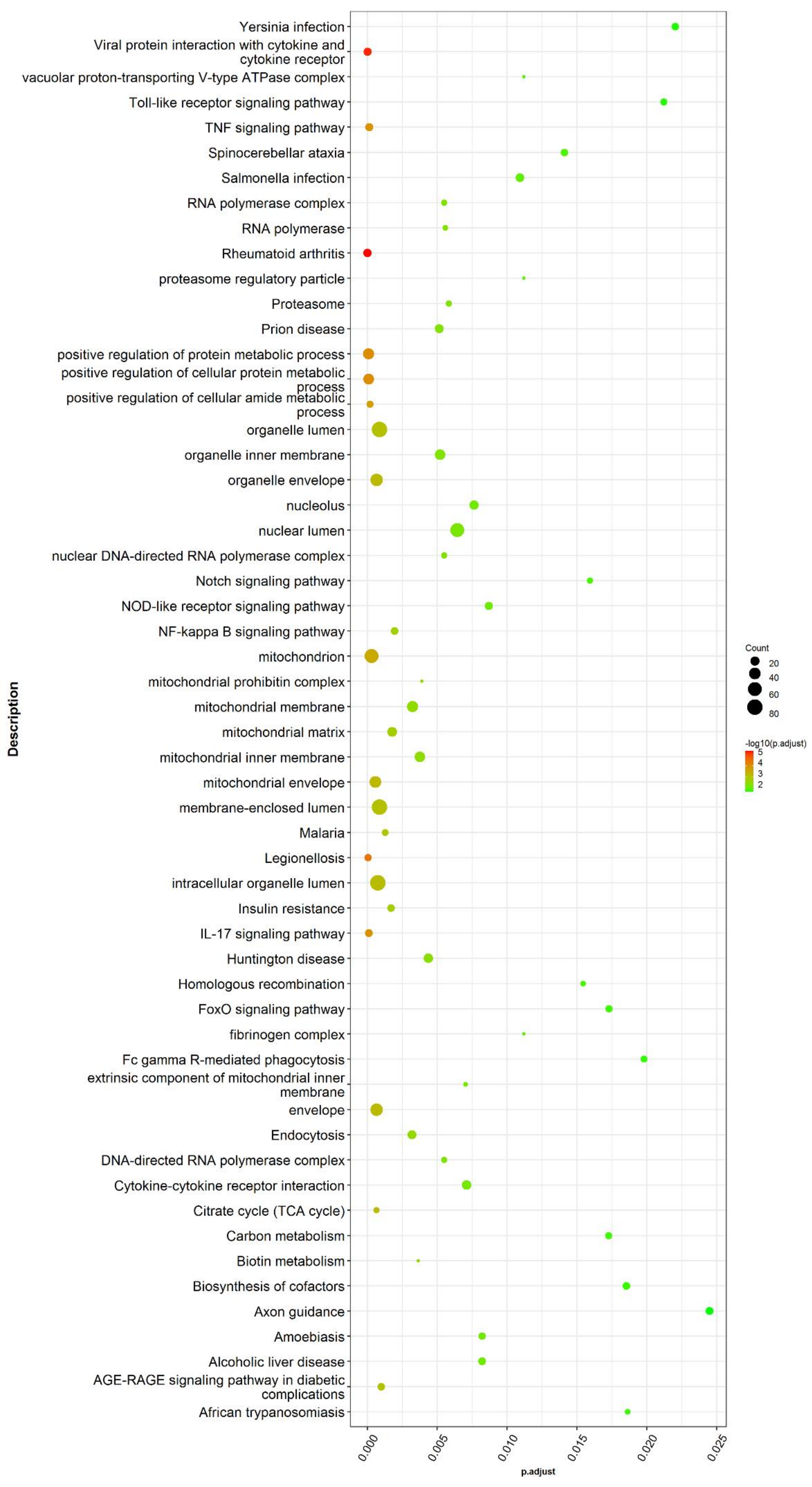

Yersinia infection
Viral protein interaction with cytokine and cytokine receptor
vacuolar proton-transporting V-type ATPase complex
Toll-like receptor signaling pathway
TNF signaling pathway
Spinocerebellar ataxia
Salmonella infection
RNA polymerase complex
RNA polymerase
Rheumatoid arthritis
proteasome regulatory particle
Proteasome
Prion disease
positive regulation of protein metabolic process
positive regulation of cellular protein metabolic process
positive regulation of cellular amide metabolic process
organelle lumen
organelle inner membrane
organelle envelope
nucleolus
nuclear lumen
nuclear DNA-directed RNA polymerase complex
Notch signaling pathway
NOD-like receptor signaling pathway
NF-kappa B signaling pathway
mitochondrion
mitochondrial prohibitin complex
mitochondrial membrane
mitochondrial matrix
mitochondrial inner membrane
mitochondrial envelope
membrane-enclosed lumen
Malaria
Legionellosis
intracellular organelle lumen
Insulin resistance
IL-17 signaling pathway
Huntington disease
Homologous recombination
FoxO signaling pathway
fibrinogen complex
Fc gamma R-mediated phagocytosis
extrinsic component of mitochondrial inner membrane
envelope
Endocytosis
DNA-directed RNA polymerase complex
Cytokine-cytokine receptor interaction
Citrate cycle (TCA cycle)
Carbon metabolism
Biotin metabolism
Biosynthesis of cofactors
Axon guidance
Amoebiasis
Alcoholic liver disease
AGE-RAGE signaling pathway in diabetic complications
African trypanosomiasis
Description
0.000
0.005
0.010
0.015
0.020
0.025
p.adjust
Count
20
40
60
80
-log10(p.adjust)
5
4
3
2

Supplementary Figure S6.

It is bubble plot of functional enrichment analysis of genes in brown module. The dot size represents the number of genes in the pathway and the color represents the pathway significance.

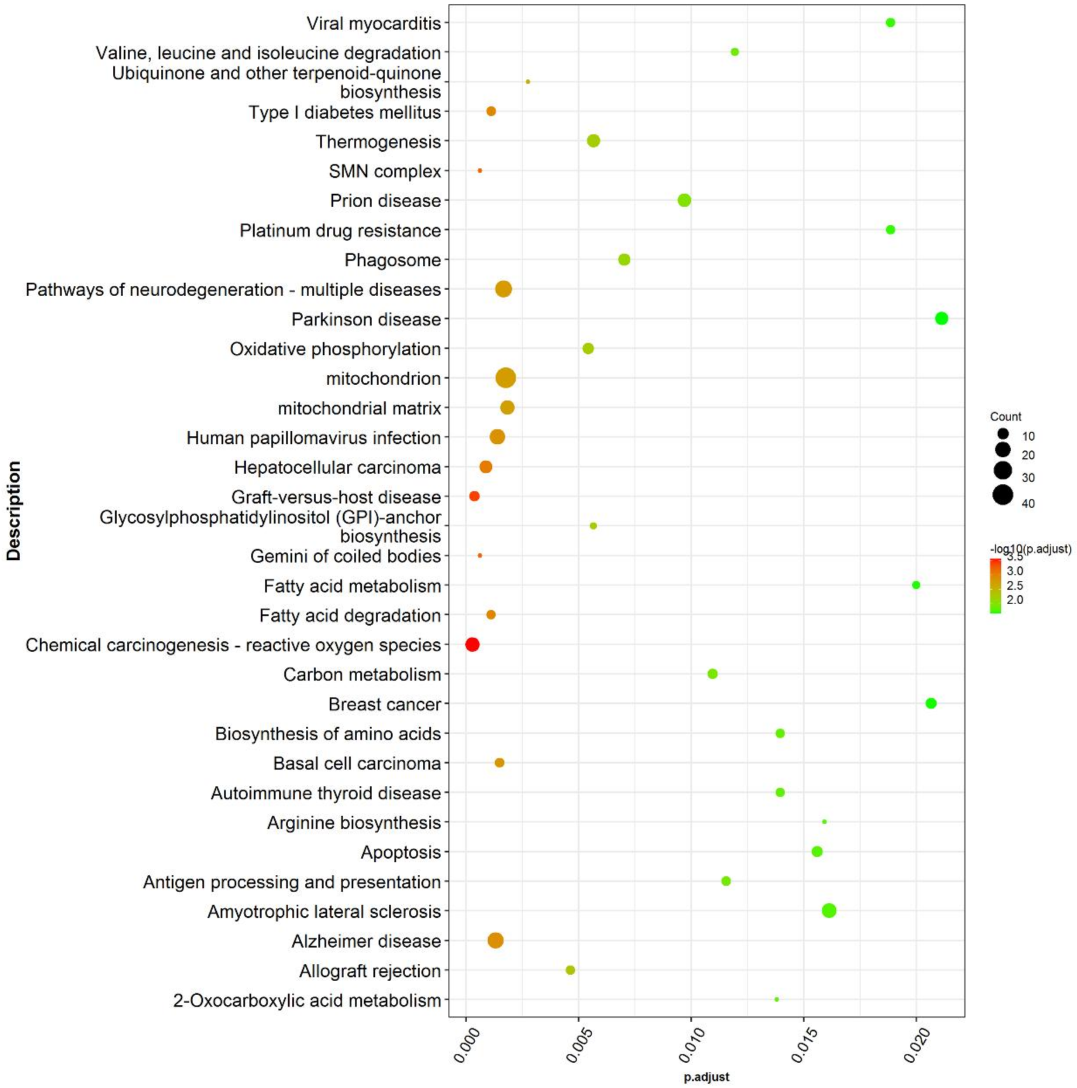

Supplementary Figure S7.

It is bubble plot of functional enrichment analysis of genes in green module. The dot size represents the number of genes in the pathway and the color represents the pathway significance.

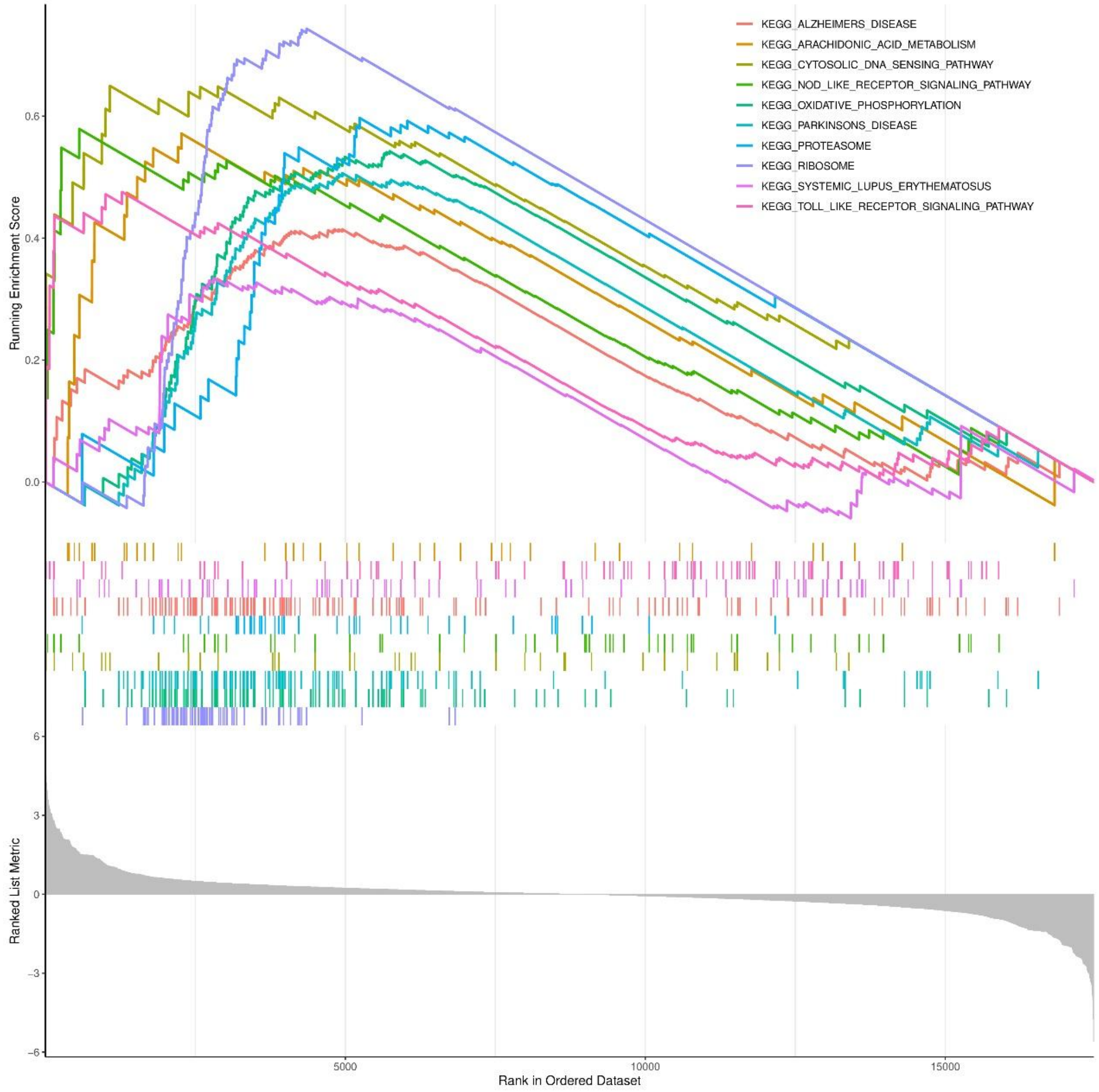

Supplementary Figure S8.

Gene set enrichment analysis (GSEA). 10 significant pathways enriched based on FDR<0.05.
